# Isoegomaketone from *Perilla frutescens* (L.) Britt Stimulates MAPK/ERK Pathway in Human Keratinocyte to Promote Skin Wound Healing

**DOI:** 10.1155/2021/6642606

**Published:** 2021-02-10

**Authors:** Ye-Ram Kim, Bomi Nam, Ah-Reum Han, Jin-Baek Kim, Chang Hyun Jin

**Affiliations:** Advanced Radiation Technology Institute, Korea Atomic Energy Research Institute, Jeongeup, Jeollabuk-do 56212, Republic of Korea

## Abstract

Skin wound healing is essential for recovery from injury, and delayed or impaired wound healing is a severe therapeutic challenge. Keratinocytes, a major component of the epidermis, play crucial roles in reepithelialization during wound healing including cell proliferation. Recent studies have shown that compounds from natural products have candidates for healing skin injury. Isoegomaketone (IK), isolated from leaves of *Perilla frutescens* var. *crispa* (Lamiaceae), has various bioactivities. However, the effect of IK on cutaneous wound healing processes has not been studied yet. In this study, we demonstrated that IK exhibits therapeutic wound healing effects using the human keratinocyte cell line HaCaT. Notably, IK promoted cell proliferation and migration in a dose-dependent manner in vitro, and treatment with 10 *μ*M IK upregulated these processes by approximately 1.5-fold after 24 h compared with the control. IK induced the activation of the MAPK/ERK pathway and cell cycle progression to the S and G2/M phases. Thus, this study demonstrates IK as a potential candidate to upregulate wound healing that may provide therapeutic benefits to patients with delayed wound healing.

## 1. Introduction

Skin is the largest organ and the first line of defense against the effects of external events, such as injury, pathogenic infection, and exposure to harmful contaminants [1]. Hence, skin wound healing is essential to resolve injury in clinical care. The cutaneous healing process consists of consecutive programmed stages, which are as follows: hemostasis, inflammation, proliferation, and remodeling [2, 3]. This physiological process requires the harmony of various factors, such as many cell types (platelets, fibroblasts, macrophages, keratinocytes, endothelial cells) and their products (cytokines, chemokines, growth factors, hormones) [4, 5]. The correlations between various diseases, such as metabolic diseases, and deficient wound healing have been well established; for instance, inadequate healing results in chronic wounds and additional infections, leading to serious clinical problems in patients with metabolic disorders, such as obesity, diabetes, and aging-related diseases. Complications in wound healing that result from these conditions can cause pain, prolong the period of illness and treatment, and increase the cost of healthcare [4, 6]. Thus, clinically effective, cost-efficient, and safe wound therapeutics strategies are urgently needed.

Keratinocytes, the major mediators of re-epithelialization responses, are key players in the proliferative phase to regenerate the epidermis and promote wound closure [7]. Keratinocytes at the edge of a wound are activated by cytokines and growth factors, such as epidermal growth factor (EGF), transforming growth factor-beta (TGF-*β*), keratinocyte growth factor (KGF), Interleukin-1 (IL-1), and tumor necrosis factor-alpha (TNF-*α*) [8–11]. Thus, keratinocytes are considered an effective therapeutic target for wound healing.

Mitogen-activated protein kinase (MAPK) signaling is essential for fundamental cellular mechanisms such as proliferation, differentiation, and migration. MAPK families are highly conserved and comprise three kinases: classical MAPK (extracellular signal-regulated kinase, ERK), C-Jun N-terminal kinase/stress-activated protein kinase (JNK/SAPK), and p38 kinase [12]. In terms of cell proliferation and migration through cellular stimulators such as growth factors, MAPK/ERK signaling is the most well-characterized among MAPK family kinases. Moreover, stimulation of the MAPK/ERK signaling pathway is considered a potential therapeutic approach for wound healing [13–16].

Natural products and botanical drugs have been traditionally used as healing substances to assist wound healing and treat skin diseases since prehistoric times [17, 18]. Numerous studies have been performed to find appropriate alternative remedies, derived from natural products, to treat wounds [19]. *Perilla frutescens* (L.) Britt is an annual herbal plant belonging to the Lamiaceae family, which has been used as a botanical drug to treat disorders such as depression-related diseases, anxiety, asthma, chest stuffiness, nausea, fever, headache, constipation, and indigestion. Current studies have demonstrated that it has pharmacological properties such as antioxidant, antibacterial, antifungal, anti-allergic, anti-depressant, anti-inflammatory, and antitumor activities [20]. Our research team has developed a functional mutant using *γ*-ray cultivar of *P. frutescens* var. *crispa* (cv. Antisperill), which exhibits high anti-inflammatory activity compared to the original cultivar. Antisperill has a high content of anti-inflammatory component, phytochemical isoegomaketone (IK). Previous studies have demonstrated that IK has clinical beneficial effects such as anti-inflammatory, anticancer, and antioxidant activities [21, 22]. However, skin wound healing effects of IK have not been reported yet.

In this study, we identified the effects of IK on wound healing using the human keratinocyte cell line, HaCaT. IK showed significant effects on cell proliferation and migration through ERK activation. The cell proliferation promoting effects of IK were blocked by the ERK inhibitor, PD98059. IK also enhanced the ratio between S and G2/M phases, promoting entry into ratio the cell proliferative phase in HaCaT cells.

## 2. Materials and Methods

### 2.1. Chemicals and Reagents

IK was isolated from the supercritical carbon dioxide (SC-CO_2_) extract, a radiation-induced mutant cultivar of *Perilla frutescens* var. *crispa* [23]. Specific antibodies for p44/42 MAPK (ERK1/2), phospho (p)-p44/p42 MAPK (ERK1/2) (Thr202/Tyr204), SPK/JNK, p-JNK (Thr183/Tyr185), p38 MAPK, and p-p38 MAPK (Thr180/Tyr182) were purchased from Cell Signaling Technology (Cell Signaling Technology, USA). Anti-*β*-tubulin antibody was obtained from Santa Cruz Biotechnology (Santa Cruz, USA).

### 2.2. Cell Culture

The human keratinocyte cell line HaCaT was cultured in Dulbecco's Modified Eagle's Medium (DMEM, Hyclone, USA) containing 10% fetal bovine serum (FBS, Gibco, USA) and 1% penicillin-streptomycin (Gibco, USA) at 37°C in a 5% CO_2_ atmosphere.

### 2.3. Cell Viability Assay

HaCaT cells (2 × 10^4^ cells/well) were seeded in 96-well cell culture plates (SPL, Korea) containing DMEM with 10% FBS and 1% penicillin and streptomycin at 37°C in an 5% incubator with 5% CO_2_ atmosphere. The cells were treated with various concentrations (0–100 *µ*M) of IK for 24 h. Cell viability was measured using the Ez-Cytox assay kit (Daeil, Seoul, Korea), according to the manufacturer's protocol:

(1) $$\begin{array}{c} \text{cell viability}\left(\%\right)=\frac{{\text{sampleOD}}_{450}-{\text{blankOD}}_{450}}{{\text{controlOD}}_{450}-{\text{blankOD}}_{450}}\;\times \;100. \end{array}$$

### 2.4. In Vitro Wound Healing Assay

HaCaT cells (5 × 10^5^ cells/well) were seeded in a 12-well plate and cultured to an almost confluent cell monolayer. A linear wound was generated in a HaCaT cell monolayer using a sterile 20–200 *µ*l pipette tip vertically. Cellular debris was removed by washing with phosphate-buffered saline (PBS, Gibco, USA). The HaCaT cells were incubated with various concentrations of IK (5, 10, and 20 *µ*M) for 24 h at 37°C in an incubator with 5% CO_2_ atmosphere. To measure wound healing ability, the scratched areas were photographed to determine the relative wound closure at 0 and 24 h after treatment. The data were analyzed using the Image J program to obtain the percentage of scratch closure at each dose of IK relative to the control.

### 2.5. Western Blot

The cells were lysed with RIPA buffer (25 mM Tris-HCl pH7.6, 150 mM NaCl, 1% NP40, 1% sodium deoxycholate, and 0.1% SDS) containing protease inhibitor cocktail and phenylmethylsulfonyl fluoride (PMSF). The concentration of the protein lysate was quantified by Bradford assay, using Bradford reagent (Bio-Rad, USA). Equal amounts of protein samples (50 *μ*g) were separated by 10% SDS-PAGE and transferred to a polyvinylidene fluoride (PVDF) membrane, which was blocked with 5% bovine serum albumin (BSA, Sigma, USA) for 1 h at room temperature and then incubated overnight at 4°C with 1 : 1000 dilutions of the appropriate primary antibodies (p38, p-p38, JNK, p-JNK, ERK1/2, and p-ERK1/2) from Cell Signaling Technology (USA) and 1 : 500 dilution of *β*-tubulin antibody from Santa Cruz Biotechnology (USA), followed by incubation with 1 : 10000 dilution of the secondary Ab at room temperature. After each incubation, the membranes were washed with TBS-Tween 20. To visualize the signals, the membranes were incubated with enhanced chemiluminescence (ECL) solution and developed using ChemiDoc Imaging System (Invitrogen, iBright CL1000, USA).

### 2.6. Cell Cycle Analyses

Cell cycle analyses were performed using a Muse Cell Cycle Kit (Merck, USA) following the manufacturer's protocol. HaCaT cells (4 × 10^5^ cells/well) were cultured in six-well cell culture plates and treated with IK (1, 5, 10 *µ*M) for 24 h. The proportion of cells in a particular phase of the cell cycle was measured using a Muse Cell Analyzer (Merck, USA) according to the manufacturer's protocol.

### 2.7. Statistical Analyses

Results are presented as mean ± SD. Statically significant differences were calculated with one-way ANOVA with Tukey's post hoc test using Prism 5.

## 3. Results

### 3.1. IK Enhanced the Proliferation of Keratinocytes

**Figure 1:**
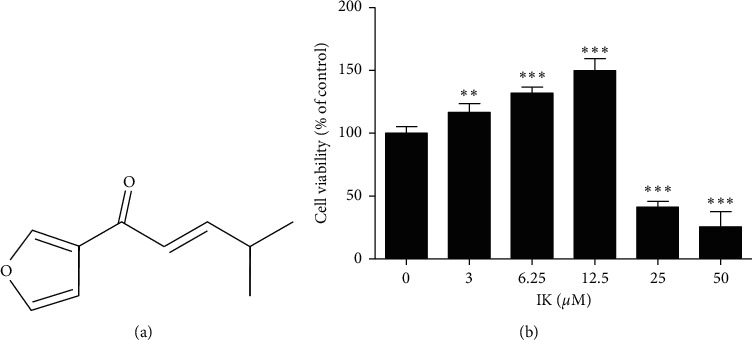
Effects of IK on cell proliferation in HaCaT cells. (a) Chemical structure of IK. (b) Cell viability of HaCaT cells with various concentrations of IK at 24 h. Cell viability was measured using an Ez-Cytox Kit. The results are presented as the means ± SD of six independent experiments ( ^*∗*^*p* < 0.05,  ^*∗∗*^*p* < 0.01, and  ^*∗∗∗*^*p* < 0.001 versus 0 *μ*M group).

Keratinocytes play essential roles in wound repair processes and proliferation and migration are important in re-epithelialization. To identify the role of IK in skin wound healing, we used the human keratinocyte cell line HaCaT [24, 25]. To study the proliferation effect of IK on human keratinocytes, cytotoxicity assays were performed. HaCaT cells were treated with various concentrations of IK for 24 h and then assayed. The chemical structure of IK is shown in Figure 1(a). At concentrations below 12.5 *μ*M, IK did not exhibit cytotoxicity, but rather upregulated cell viability at 3–12.5 *μ*M compared with 0 *μ*M IK at 24 h following treatment (Figure 1(b)). Thus, we selected IK concentrations <10 *μ*M to investigate the effects of IK on cell proliferation. The results indicated that IK enhanced cell proliferation in HaCaT cells and that IK induced potent cellular proliferation at low concentrations (below 12.5 *μ*M).

### 3.2. IK Induced Cell Migration of Keratinocytes In Vitro

**Figure 2:**
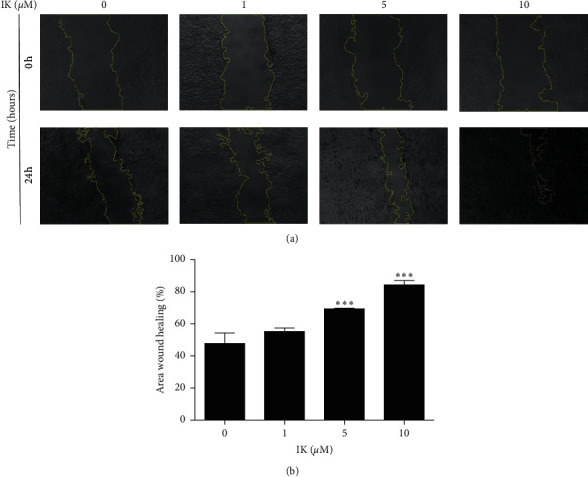
Effects of IK on scratch wound closure of HaCaT cells in vitro. HaCaT cell monolayer was mechanically scratched and incubated with the indicated concentration of IK. HaCaT cell migration was monitored using a microscope at 0 and 24 h after scratch. (a) Images of HaCaT cells treated IK at 0 and 24 h at 10 × magnification. The yellow line shows the border of the wound. (b) Relative wound closure area of HaCaT cells according to IK concentration. The wound area was measured using Image J and calculated as the ratio of the scratch area at 24 h relative to 0 h was calculated. The results are presented as the means ± SD of three independent experiments ( ^*∗*^*p* < 0.05,  ^*∗∗*^*p* < 0.01, and  ^*∗∗∗*^*p* < 0.001 versus 0 *μ*M group).

In cutaneous wound healing processes, keratinocytes migrate inward from the edges of the wound to restore the integrity of the skin barrier. To test whether IK affects cell migration, we performed a scratch wound assay in HaCaT cells. After scratching, HaCaT cells were incubated with 1, 5, and 10 *μ*M for 24 h. The scratch area was photographed and measured, revealing that IK significantly decreased the wound area in a dose-dependent manner. These data show that IK enhances cell migration in HaCaT cells (Figure 2).

### 3.3. IK Promoted the Proliferation of Keratinocytes through the Activation of the ERK Pathway

**Figure 3:**
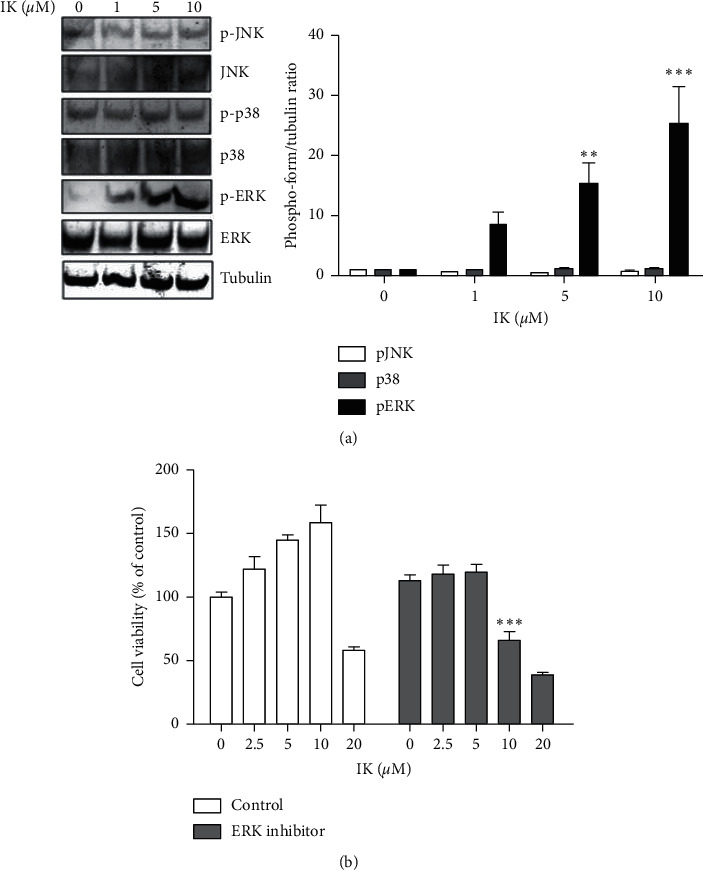
IK enhances proliferation of keratinocytes through induction of ERK 1/2 pathway. (a) Western blot of HaCaT cells treated with varying concentrations of IK. HaCaT cells were treated with IK (0, 1, 5, and 10 *μ*M) for 24 h. Whole-cell extracts were harvested and analyzed using the antibody against total JNK, ERK1/2, p38, phospho-JNK, ERK1/2, p38, and beta-tubulin was used as the loading control. (b) Viability of HaCaT cells compared with only IK treatments and cotreated with ERK 1/2 inhibitor, PD98059. HaCaT cells were seeded into a 96-well cell culture plate and treated with various concentrations of IK only or with ERK 1/2 inhibitor for 24 h. Cell viability was measured using an Ez-Cytox Kit. The results are presented as the means ± SD of three independent experiments ( ^*∗*^*p* < 0.05,  ^*∗∗*^*p* < 0.01, and  ^*∗∗∗*^*p* < 0.001 versus control group).

As IK induced cell proliferation and migration, we further investigated the possible underlying molecular mechanism. MAPK family kinases, including classical MAPK (also known as ERK), c-Jun N-terminal kinase/stress-activated protein kinase (JNK/SAPK), and p38 kinase, play essential roles in the regulation of cellular proliferation [12]. We hypothesized that IK induces cell proliferation and migration through the activation of the MAPK pathway. To verify the hypothesis, we treated HaCaT cells with IK for 24 h and compared the phosphorylated forms of MAPK family kinases. IK dramatically enhanced the level of phospho-ERK, whereas phospho-JNK and p38 were unaffected by IK treatment (Figure 3(a)). Next, we hypothesized that IK regulates cell proliferation and migration through activation of the ERK pathway. So, we used the ERK inhibitor, PD98059, to confirm the role of the ERK pathway in the effects of IK on the proliferation of HaCaT cells. The ERK inhibitor blocked the effect of IK on cell proliferation. The group treated with IK and ERK inhibitor showed significant cell toxicity compared to the group treated with IK alone, showing an especially large difference at 10 *μ*M (Figure 3(b)). Taken together, these results indicate that IK promotes cell proliferation through the activation of the ERK pathway in vitro.

### 3.4. IK Promoted Cell Cycle Progression

**Figure 4:**
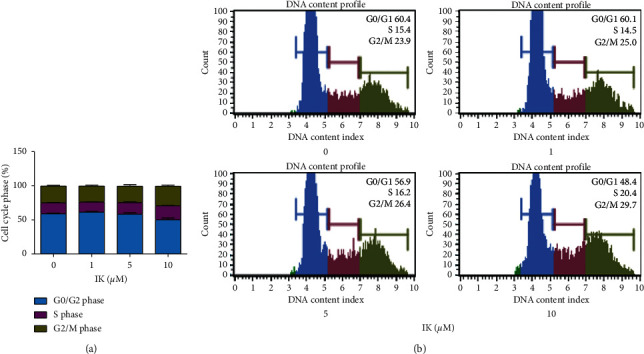
Effects of IK on cell cycle progression in HaCaT cells. (a) Cell cycle ratio of HaCaT cells after treatment with IK. (b) A plot of the cell cycle pattern of HaCaT cells under different treatment concentrations of IK. The results are presented as the means ± SD of three independent experiments.

The Ras/Raf/MEK/ERK signaling pathway is well known as a regulator of key biological responses such as cell growth, proliferation, differentiation, and survival [26]. The ERK family is required to enter the cellular mitogenesis stage as a cell cycle checkpoint for DNA replication [27]. We presumed that IK could be a cell growth simulator that induces progression from the G0/G1 phase to S phase in wound repair processes in keratinocytes. To demonstrate this, we treated HaCaT cells with IK for 24 h and then analyzed the cell cycle of HaCaT cells. We found that the proportion of cells in the S phase increased with IK concentration in a dose-dependent manner. Moreover, proportion of cells in the G2/M phase also increased in the IK treatment group (Figure 4). These results showed that IK acts as a growth stimulator and increases keratinocyte proliferation and cell cycle progression.

## 4. Discussion

In this study, we found that IK induced the proliferation of the HaCaT cells. Based on this result, we analyzed the effects of IK on cell migration. In an in vitro scratch assay, IK promoted cell wound closure in a dose-dependent manner at 24 h. To clearly understand the underlying mechanism, we investigated the effects of IK on the activation of MAPK signaling. IK induced ERK signaling pathway and enhanced cell proliferation; however, this effect was diminished through ERK pathway inhibition using an inhibitor. Additionally, we found that IK acts as a growth factor to induce cell cycle progression. Taken together, these results demonstrate that IK affects skin wound healing through the MAPK/ERK pathway.

Inadequate wound healing is a clinically urgent problem that needs to be addressed. Since wound healing depends on host status, it is usually poor in elderly people and patients with metabolic disorder [28, 29]. In particular, wound repair is an important part of recovery after surgery or radiation therapy [30]. Moreover, wound healing process is a complex process involving many cell types. This study confirmed the effects of IK on wound healing in vitro. A previous study showed that *P. frutescens* Britton leaf extract inhibited cell growth, migration, and adhesion of human cancer cells, including HCT116 colorectal carcinoma cells and H1299 non-small-cell lung carcinoma cells [31]. Therefore, the effects of IK on other cell types should be confirmed to determine its potential for use in the treatment of patients. Since the process of re-epithelialization is a combination of various factors, additional studies involving *in vivo* models are required to confirm the outcome of the overall effects of IK on wound healing. This in vitro assay may be a cornerstone for further studies.

Another research strategy to identify candidates for skin disorder therapy is to confirm the anti-inflammatory effects of the therapeutic compounds in skin conditions [32, 33]. Perilla essential oil components have been reported as effective anti-inflammatory agents [34, 35]. Our group has previously reported the anti-inflammatory activities of IK on RAW264.7 macrophage cells [36]. However, IK treatment did not show significant anti-inflammatory effects in TNF-*α*- and IFN-*γ*- stimulated HaCaT cells (data not shown). These results show that the dominant effects of IK on keratinocytes are associated with cell proliferation, not anti-inflammatory effects.

The MAPK/ERK pathway, also known as Ras-Raf-MEK-ERK pathway, is essential for the regulation of cell proliferation. Growth factors and cytokines stimulate this pathway and the signal triggers downstream responses. This pathway is known as a cellular checkpoint in cellular mitogenesis [12]. We confirmed that IK induced the cell cycle to progress from the G0/G1 (gap1) phase to the S (Synthesis) and G2 (gap2)/M (mitosis) phases (Figure 4). Cyclins are a family of proteins that regulate cell cycle progression by activating cyclin-dependent kinases (CDKs) [37]. Given the significance of the cyclin pathway in wound healing, the effects of IK on the regulation of cyclin families, especially cyclin D1 regulated by the MAPK/ERK pathway, should be investigated [38, 39]. These results suggested that IK stimulates the MAPK/ERK pathway in keratinocytes, which is crucial in wound healing processes.

Alternative medicines derived from medicinal plant extracts have been used to treat diseases for a long time [40]. *Perilla frutescens,* a representative medical plant, has been used to treat various diseases, particularly in East Asian countries such as Korea, China, Japan, and India. In particular, its leaves contain a variety of compounds, which have been used as functional dietary supplements [20]. IK is a major volatile component of *P. frutescens* leaves. We have previously developed IK as a dietary supplement for the treatment of rheumatoid arthritis [41].

In this study, we hypothesized that IK could be a promising therapeutic agent in alternative medicine for diseases with symptoms of compromised wound healing, such as diabetes, obesity, and aging. IK shows potent proliferation promoting effect in keratinocytes at low concentrations, but exhibits strong cytotoxicity at high concentrations (>20 *μ*M) (Figure 1(b)), as compared to other compounds derived from natural products [25, 42]. Thus, the appropriate concentration and administration methods of IK are important for its application to animal models or patients.

Overall, these findings indicate that IK induces MAPK/ERK pathways in keratinocytes, leading to increased proliferation and rates of migration, both crucial to wound healing. *Perilla frutescens* contains the active ingredient IK and may have a medicinal role in treating patients with delayed wound healing.

## 5. Conclusions

In conclusion, we demonstrated that IK isolated from *P. frutescens* induced cell proliferation and migration of the human keratinocytes through the activation of the MAPK/ERK1/2 pathway. This study suggests that IK has a wound healing effect similar to a growth factor; thus, it might be a potential pro-regenerative agent for impaired wound healing.

## Data Availability

The data used to support the findings of this study are available from the corresponding author upon request.
